# Enhanced Photocatalytic Activity of Cu_2_O Cabbage/RGO Nanocomposites under Visible Light Irradiation

**DOI:** 10.3390/polym13111712

**Published:** 2021-05-24

**Authors:** Appusamy Muthukrishnaraj, Salma Ahmed Al-Zahrani, Ahmed Al Otaibi, Semmedu Selvaraj Kalaivani, Ayyar Manikandan, Natarajan Balasubramanian, Anwar L. Bilgrami, Mohamed A. Riswan Ahamed, Anish Khan, Abdulaah M. Asiri, Natesan Balasubramanian

**Affiliations:** 1 Department of Chemistry, C.B.M. College (Affiliated to Bharathiar University), Coimbatore 641 042, Tamil Nadu, India; muthukrishnaraj@gmail.com (A.M.); kalaiche05@gmail.com (S.S.K.); 2 Chemistry Department, Faculty of Science, University of Ha’il, P.O. Box 2440, Ha’il 81451, Saudi Arabia; s.alzahrane@uoh.edu.sa (S.A.A.-Z.); ahmed.alotaibi@uoh.edu.sa (A.A.O.); 3 Department of Chemistry, Bharath Institute of Higher Education and Research (BIHER), Bharath University, Chennai 600 073, Tamil Nadu, India; manikandana.che@bharathuniv.ac.in; 4 Centre for Nanoscince and Nanotechnology, Bharath Institute of Higher Education and Research (BIHER), Bharath University, Chennai 600 073, Tamil Nadu, India; 5 Department of Chemistry, SRM Institute of Science and Technology, Faculty of Engineering and Technology, Ramapuram Campus, Bharathi Salai, Ramapuram, Chennai 600 089, Tamil Nadu, India; balabhctry@gmail.com (N.B.); polyrizwan@gmail.com (M.A.R.A.); 6 Deanship of Scientific Research, King Abdulaziz University, Jeddah 21589, Saudi Arabia; bilgrami1956@hotmail.com; 7 Center of Excellence for Advanced Materials, King Abdulaziz University, Jeddah 21589, Saudi Arabia; anishkhan97@gmail.com (A.K.); asiri2@kau.edu.sa (A.M.A.); 8 Chemistry Department, Faculty of Science, King Abdulaziz University, Jeddah 21589, Saudi Arabia; 9 Electrochemical Engineering Lab, Department of Chemical Engineering, A.C. Tech Campus, Anna University, Chennai 600 025, Tamil Nadu, India

**Keywords:** graphene/Cu_2_O, nanocomposite, Cu_2_O cabbage, photocatalyst, dye degradation

## Abstract

Towards the utilization of Cu_2_O nanomaterial for the degradation of industrial dye pollutants such as methylene blue and methyl orange, the graphene-incorporated Cu_2_O nanocomposites (GCC) were developed via a precipitation method. Using Hummers method, the grapheme oxide (GO) was initially synthesized. The varying weight percentages (1–4 wt %) of GO was incorporated along with the precipitation of Cu_2_O catalyst. Various characterization techniques such as Fourier-transform infra-red (FT-IR), X-ray diffraction (XRD), UV–visible diffused reflectance (UV-DRS), Raman spectroscopy, thermo gravimetric analysis (TGA), energy-dispersive X-ray analysis (EDX), and electro chemical impedance (EIS) were followed for characterization. The cabbage-like morphology of the developed Cu_2_O and its composites were ascertained from field-emission scanning electron microscopy (FESEM) and high-resolution transmission electron microscopy (HR-TEM). In addition, the growth mechanism was also proposed. The results infer that 2 wt % GO-incorporated Cu_2_O composites shows the highest value of degradation efficiency (97.9% and 96.1%) for MB and MO at 160 and 220 min, respectively. Further, its catalytic performance over visible region (red shift) was also enhanced to an appreciable extent, when compared with that of other samples.

## 1. Introduction

The contamination of carcinogenic organic dyes in water bodies is the serious environmental problem in the world. Especially, it is a potential threat to soil, water, plant and human being, when discharging of dyestuffs without proper treatment [1,2,3]. Synthetic dyes play an important role in many industries, but it is hardly difficulty to determine the quantity of dyes discharged in various industrial processes. It is reported that the more than 100,000 synthetic dyes are commercially available in market and their major application is in the field of textile printing industry to color fabrics [4,5]. The wastewater coming out from these industries to water resources causes unavoidable health issues such as nausea, vomiting, headache, and high blood pressure [6]. Dyes also block the sunlight penetration in water, which increases the COD and thereby affects the photosynthetic activities and growth of bacteria [7].

The utilization of natural color for material coloring has been practiced since the most recent 5000 years. The invention of synthetic dyes becomes stated in 19th century, which has suppressed the usage of natural dye. The artificial dyes may be produced in large part and may be utilized in various industries. Dyes are in three different categories specifically (i) anionic dyes, (ii) cationic dyes, and (iii) non-ionic dyes [8]. Reactive and primary dyes are extensively used in the fabric enterprise due to their traits of water-soluble nature, shiny coloration, cheaper to produce and easier to apply on fabrics.

Methylene blue (MB) has complex structure and it is a usually utilized color in the apparel business as well as in various synthetic, clinical, and natural applications. Because of its complex structure, it makes more troubles in the environment [9]. Likewise, MO is used as a water-soluble azo dye and even at very low concentrations, which causes unfavorable outcomes on photosynthesis in water bodies [10].

Membrane treatment [11], adsorption [12], fenton oxidation [13], electrochemical [14], biological [15], photocatalysis [16], and other methods and technologies are actively attempted for efficient remediation of dyes in the effluents. Polymers and polymer inorganic nanocomposite are also an excellent recent material to remove contamination of industrials waste because of the high surface area of the polymer and its composite with inorganic metal oxides. Polymer inorganic nanocomposite are being used for desalination and metal ion sensing currently. Among all these methods and strategies, the photocatalytic degradation technique has emerged as a promising process for degrading dyes from wastewater as a higher capable approach due to its financially viable and ease [17]. The photocatalysis technique cannot only decolorize the wastewater but it also achieves the degradation of dyes.

Graphene is a very appealing material because of its exceptional nanostructure and remarkable properties [18]. Graphene is an ultrathin material and can act as the graphene sheets, which lead to the production of novel type of graphene-based materials [19,20,21,22].

Grapheme oxide (GO) is a proved promising material for the degradation of dyes due to its high surface area, ample functional groups, flexible sheets, unique electronic band structure, and extraordinary mechanical strength. Recently, attention has focused on the performance of metal oxide graphene nanocomposites with different concentrations, structural composite ratios, and surface features. In this regard, many researchers have made a detailed study on the performance of nanocomposites of GO–ZnO and GO–TiO_2_ [23].

In this regard, metal oxide nanocomposites have gained major attraction. Many of the diverse heterogeneous photo catalysts, copperous oxide (Cu_2_O), have been extensively used due to its photoconductive in addition to photochemical properties that have more advantages. Cupperous oxide is one of the core minerals known to be an ecofriendly semiconductor, which is less expensive, has low toxicity with high durability, and has small bandgap energy of 2.0–2.2 eV [24,25]. Hence, loading Cu_2_O on graphene oxide offers the guarantee of accomplishing the exceptionally effective photocatalysis for the removal of organic pollutants under sunlight. Despite this, graphene-based Cu_2_O could be a suitable material for photocatalytic applications, and a definite auxiliary examination of Cu_2_O-graphene has not been accounted so for.

Owing to the interesting facts about both Cu_2_O and graphene enlivened us to plan and integrate graphene-based Cu_2_O composite, which can proficiently use the combinative benefits of Cu_2_O and GO in the present work to get a photocatalyst with prevalent execution.

## 2. Experimental

### 2.1. Materials

Flakes of graphite were obtained from Alfa easer. Ba(NO_3_)_2_, Cu(NO_3_)_2_·3H_2_O, KMnO_4_, KBr, NaNO_3_, H_2_SO_4_ (98%), HCl (37%), H_2_O_2_, C_6_H_12_O_6_, NaOH, and C_2_H_5_OH were purchased from Merck, Mumbai, India. Methylene blue, methyl orange, and Degussa P25 (50 nm) were purchased from Sigma-Aldrich, Mumbai, India. All chemicals were of analytical grade, so they do not require any further purification, and deionized (DI) water was used for solution preparation.

### 2.2. RGO/Cu_2_O Composite Synthesis

**Scheme 1:**
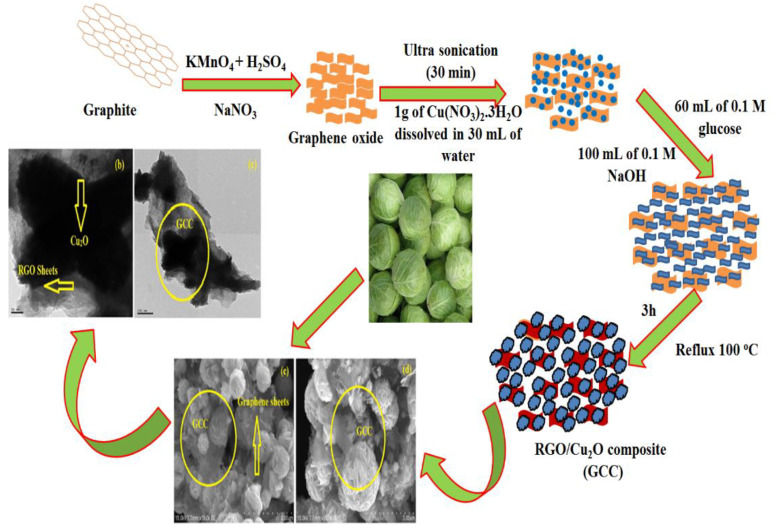
Synthesis of RGO/Cu_2_O composite (GCC).

The cabbage-like reduced graphene oxide/Cu_2_O composite (GCC) was synthesized by the precipitation method shown in Scheme 1. A pretreated graphene oxide (30 mg) and copper nitrate trihydrate Cu(NO_3_)_2_·3H_2_O (1 g) were taken and then dissolved in 30 mL distilled water with 30 min ultrasonication at room temperature.

Then, 0.1 M glucose (60 mL) and 0.1 M NaOH (100 mL) solution were added to the above mixture in dropwise under constant stirring at 100 °C. After 3 h, the RGO/Cu_2_O nanocomposite was formed as a reddish-brown precipitate. Then, it was continuously washed with DI water and ethanol. Further, it was centrifuged and dried (70 °C) in vacuum oven for 6 h to get GCC composite. The procedure was repeated again for the preparation of pure Cu_2_O without graphene oxide. Difference weight percent (1%, 2%, 3%, and 4%) of GO were utilized for the preparation of various GCC composites as GCC 1, GCC 2, GCC 3, and GCC 4.

### 2.3. Characterization

Different spectral studies were used such as XRD (Xpert-Pro-PAN), Almelo, Netherlands, FT-IR (Thermo Scientific Nicolet iS5), Mumbai, India. UV-DRS (Shimadzu UV-1800) Kyoto, Japan, Raman (RENISHAW inVia), Bangalore, India, FESEM and EDX (Tescan Vega3 (Czech Republic) Horiba HU77 (Japan)), HRTEM (FEI, TECHNAI G2 30S–twin D905(USA)), TGA (Perkin Elmer, Q500 Hi-Res) Kolkata, India, and EIS (CHI660D- Austin, TX, USA) for characterization [26].

### 2.4. Photocatalytic evaluation

Photocatalytic study was carried out from visible light irradiation (300 W Xenon lamp) with a 400 nm cutoff filter. A prepared GCC (100 mg) was dispersed in 0.01 g/L concentration of MB and MO dye solutions in 100 mL. Before starting the irradiation, the mixture was kept for 30 min at dark condition with continuous stirring. At room temperature, the photocatalytic behavior of prepared samples was determined with quartz glass photocatalytic reactor. Further, the same method was used for the catalytic behavior of pure Cu_2_O and Degussa P25.

## 3. Results and discussion

### 3.1. FT-IR Analysis

**Figure 1:**
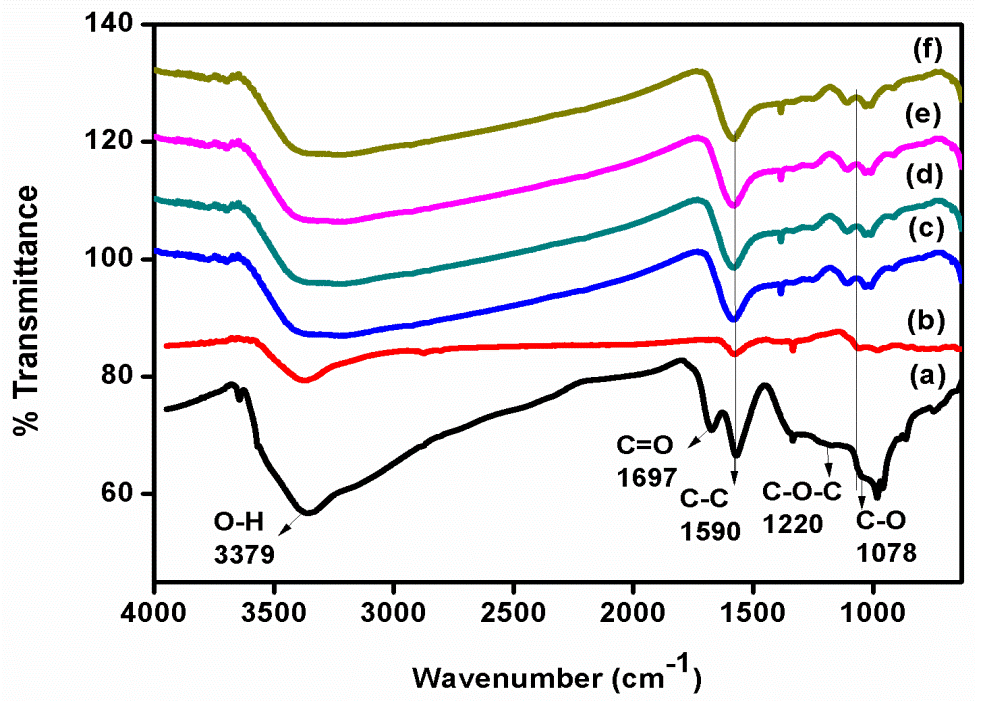
FT-IR spectra of (**a**) GO, (**b**) Cu_2_O, and (**c**–**f**) GCC 1, 2, 3 and 4.

FT-IR spectral analysis has been used to identify the reduction of oxygen-containing functional groups in GO sheets during reflux process with low temperature. The FT-IR analysis of GO, pure Cu_2_O, and GCC (1–4%) composites is shown in Figure 1. The stretching and bending vibrations of the OH group are attributed from the peaks appeared at 3379 and 1590 cm^−1^. The skeletal ring, aromatic carbon, and C=O stretching vibrations in the graphitic domain can be ascribed by the peaks appeared at 1697, 1220, and 1078 cm^−1^. The Cu_2_O skeletal vibration is attributed at peak 636 cm^−1^. Then, the disappearance of peak at 1220 cm^−1^ confirmed that glucose could reduce both Cu^2+^ and GO to produce GCC during reflux reduction process.

### 3.2. Structure Investigation

**Figure 2:**
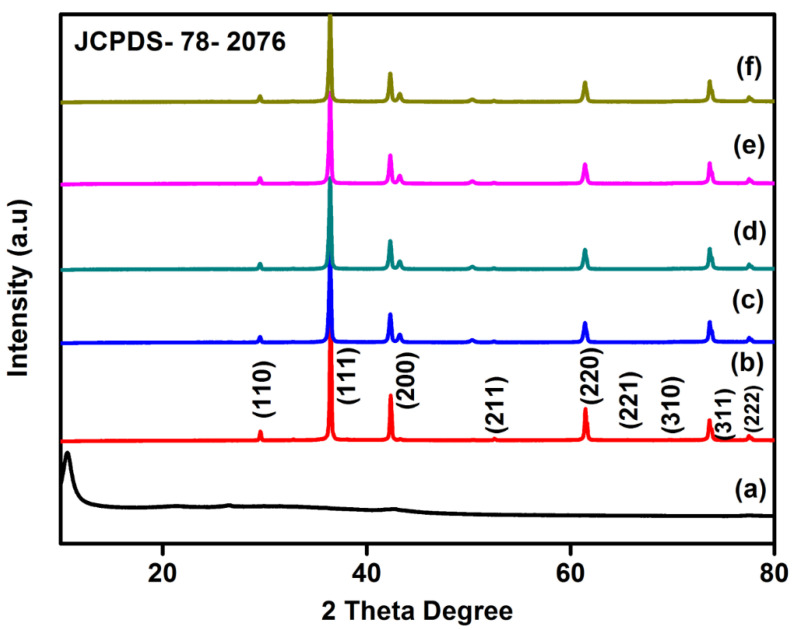
XRD of (**a**) GO, (**b**) Cu_2_O, and (**c**–**f**) GCC 1, 2, 3, and 4.

The crystalline and amorphous nature of GO, Cu_2_O, and GCC 1, 2, 3, and 4 nanocomposites were examined using XRD patterns as shown in Figure 2a–f. In the case of GO, reflection peak appeared at 10.75° and the interlayer distance between its oxide layers is found to be 0.8133 nm as shown in Figure 2a. The peaks at 29.58°, 36.44°, 42.32°, 52.48°, 61.40°, 65.58°, 69.62°, 73.55°, and 77.41° which were attributed to the (110), (111), (200), (211), (220), (221), (310), (311), and (222) planes for pure Cu_2_O. The cubic phase of Cu_2_O structure matched with JCPDS card number 78–2076 from the reflection peaks of Cu_2_O. The d-spacing was calculated as 0.24 nm with respect to plane (111) in Cu_2_O. The peaks appeared for GCC also exhibit comparable diffraction peaks and confirm the presence of Cu_2_O in the composite. Further, the formation of reduced graphene oxide (RGO) has been confirmed by the disappearance of peak 10.75° in XRD patterns of GCC composites. The reduction in Cu_2_O peak intensities further confirms the formation of RGO in composites. Because of the lower weight percentage of RGO in the composites, the expected peak at 23.9° has not been observed in the patterns of composites.

### 3.3. Raman analysis

**Figure 3:**
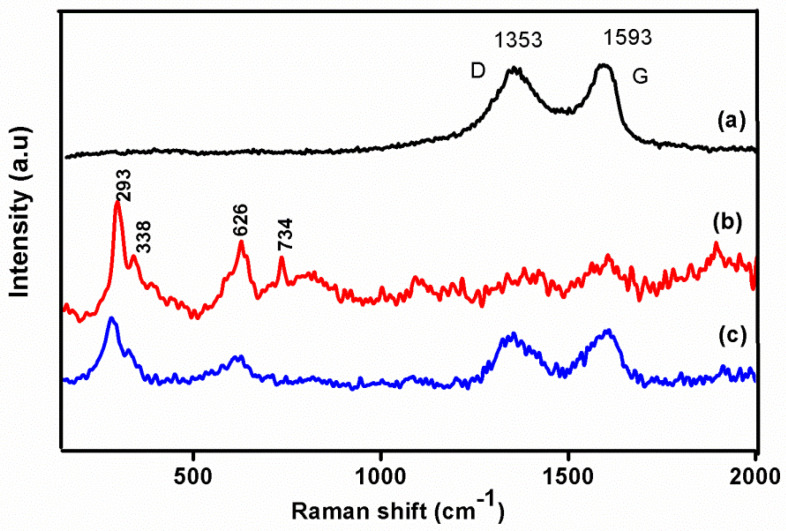
Confocal Raman spectra of (**a**) GO, (**b**) Cu_2_O, and (**c**) GCC 2.

The spectra of Raman analysis for GO, Cu_2_O, and GCC 2 are shown in Figure 3. The obtained D band (1353 cm^−1^) and G-band (1593 cm^−1^) from graphene oxide indicate the presence of sp^2^ carbon skeleton in graphene. The shifting values of D and G band indicate the reduction of graphene oxide to graphene and the interaction between the RGO and Cu_2_O matrix in GCC 2 composite.

From the spectrum of Cu_2_O, four peaks at 293, 338, 626, and 734 cm ^−1^ were exhibited and the same peaks were also found in the GCC 2 but with low intensity. This confirms the anchoring of Cu_2_O nanoparticles in the RGO. The increase in ratio between the intensity of D and G band from 0.88 in GO to 0.98 in GCC 2 confirms the reduction of oxygen functionalities in composites.

### 3.4. UV–Vis Diffuse Reflectance Spectrum Studies

**Figure 4:**
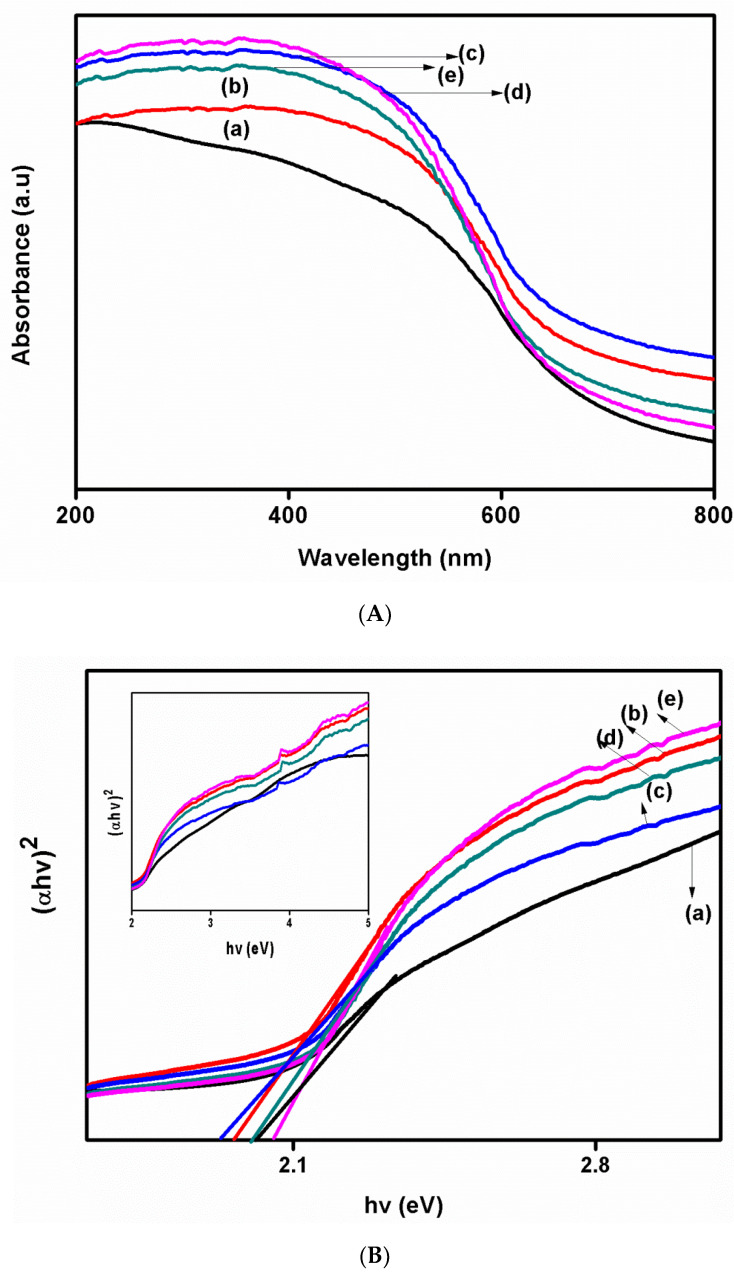
(**A**) UV-DRS spectra of (**a**) Cu_2_O and (**b**–**e**) GCC 1, 2, 3, and 4. (**B**) Band gap spectra of (**a**) Cu_2_O and (**b**–**e**) GCC 1, 2, 3, and 4.

The UV-DRS spectra of Cu_2_O and four GCC composites are revealed in Figure 4A. The peak at 650 nm denotes the presence of Cu_2_O with high crystalline nature. It is noted from the results that the absorbance of visible light region by GCC increases with red shift due the incorporation of graphene on Cu_2_O. This may also significantly influence the optical properties for the enhancement of visible-light absorption. Hence, the improved photocatalytic activity under visible-light is expected. The band-gap energies for pure Cu_2_O, GCC 1, 2, 3, and 4 are calculated to be (Figure 4B) 2.01, 1.96, 1.93, 2.00, and 2.05 eV.

### 3.5. Thermogravimetric Analysis

**Figure 5:**
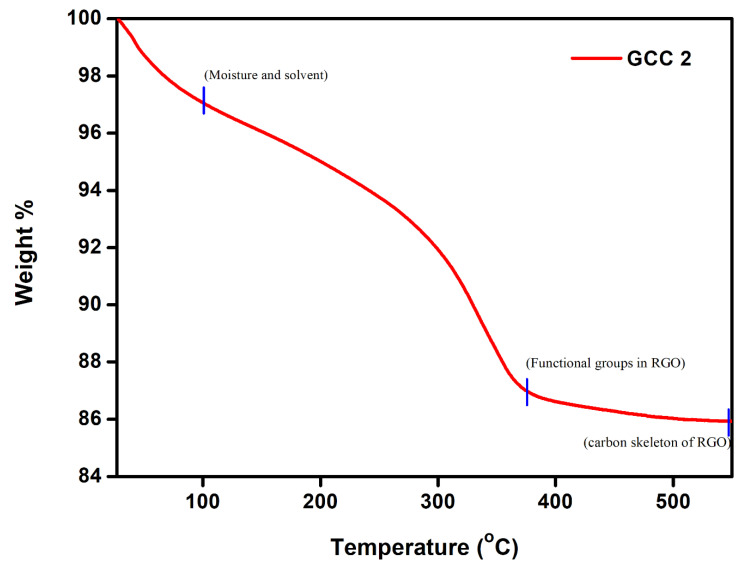
TG curve of GCC 2.

The thermogravimetric analysis of GCC 2 nanocomposite is shown in Figure 5. It is clear that there is negligible weight loss observed up to 100 °C for the presence of moisture and solvent. The loss of weight (13.2%) in the region of 100–375 °C is ascribed to the removal of residual functional groups in RGO. The loss of weight up to 14.1% in the region of 375–550 °C is endorsed to the decomposition of carbon skeleton of RGO. The observed weight loss within the particular range of temperature shows that the composite possesses good thermal stability, which also directed to perform the catalytic activity of prepared GCC 2 nanocomposite at below 500 °C.

### 3.6. FESEM and EDX Analysis

**Figure 6:**
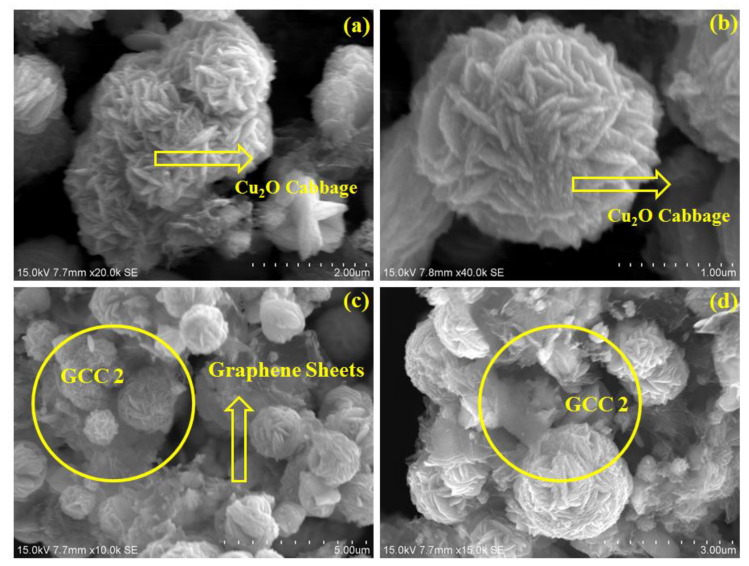
SEM pictures of (**a**,**b**) Cu_2_O and (**c**,**d**) GCC 2.

**Figure 7:**
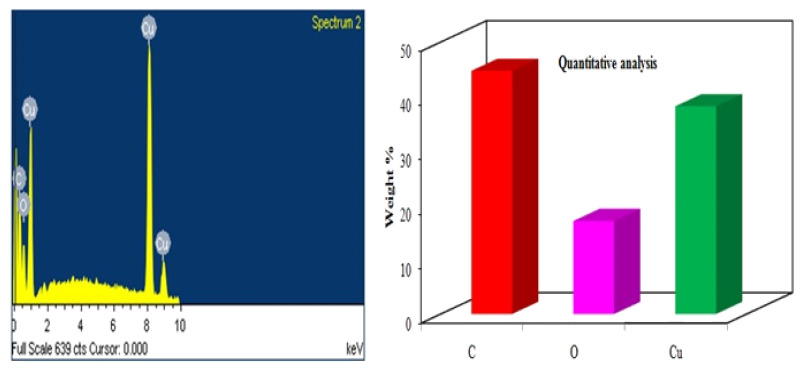
EDX spectrum of GCC 2.

In order to study about the morphology of Cu_2_O and GCC 2 composite, the FESEM analysis was carried out. In Figure 6a,b, it is found that a large number of novel cabbage-like structures are obtained from pure Cu_2_O. Figure 6c,d exhibit the interfacial contact between RGO and Cu_2_O matrixes. The RGO sheets are attached with the Cu_2_O material, which may be favorable to generate photoelectrons and thus improve the photocatalytic activity. The chemical composition of the synthesized GCC 2 composite was established from the analysis of EDX spectrum in Figure 7. It shows that the prepared GCC contains C, Cu, and O, and no other impurities were detected.

The growth mechanism of the cabbage-like Cu_2_O architecture under precipitation technique was investigated, and it follows nucleation-dissolution recrystallization mechanism with controlled kinetics, because the nanoparticles were formed in the solution phase system at 100 °C through homogeneous nucleation process [27,28]. Sodium hydroxide (NaOH) acts as a significant role for the development of cabbage-like Cu_2_O architecture. It helps to dissolute the Cu_2_O and forms the intermediate such as Cu(OH)_2_ which further leads the formation of nanoparticles via Ostwald ripening process [29]. Moreover, glucose was also considered as an influencing factor for the growth of Cu_2_O nanoparticles. The above factors help to design the structure of nanocomposites with well-defined morphologies.

### 3.7. HR-TEM Analysis

**Figure 8:**
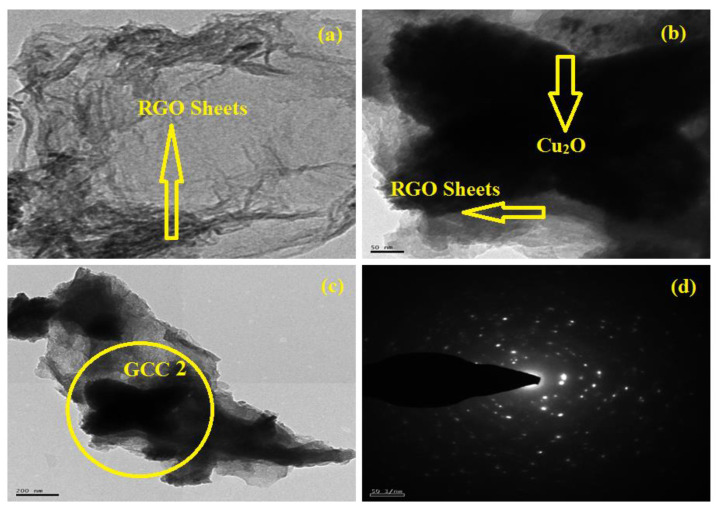
HRTEM pictures of (**a**) RGO, (**b**,**c**) GCC 2, and (**d**) SAED pattern of GCC 2.

HRTEM images of RGO and the GCC were examined and shown in Figure 8a, which showed that the surface of RGO sheet consists of many wrinkles, which helps to attach the metal particles easily on its surface. The Cu_2_O nanocabbages are anchored on the surface of the RGO sheets as shown in Figure 8b,c. From the HR-TEM images of GCC 2, it is evident that the homogenous deposition is achieved in between RGO and Cu_2_O with no aggregation. The highly dispersed nanocabbage attached on the large surface area of graphene increase the photocatalytic activity. In addition, the SAED pattern of Figure 8d further proved the incorporation of cabbage-like Cu_2_O into RGO composite and confirms the crystalline nature.

### 3.8. EIS Analysis

**Figure 9:**
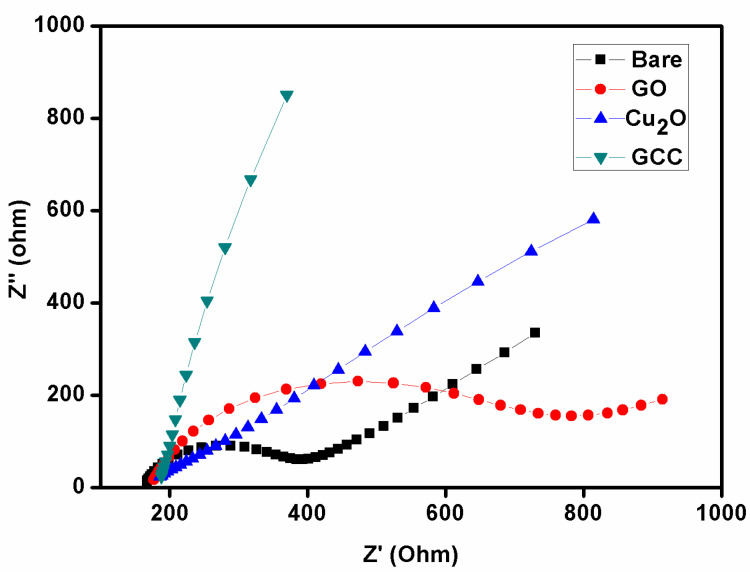
EIS spectra of Bare, GO, Cu_2_O, and GCC 2.

It is an important tool for studying the interface surface properties of electrode materials. EIS was employed to examine the fast ion transport within GCC 2 electrode materials. The spectra of bare electrode, pure GO, Cu_2_O, and GCC 2 were illustrated (Figure 9) in the range of 0.01 Hz-100 KHz frequency at open circuit potential of 5 mV using 2.5 mM Fe^2+^/Fe^3+^ solution. GCC 2 exhibits least internal charge transfer resistance (Rct) and low semicircle arc than pure Cu_2_O. This may be due to the 2D RGO, which enhance the photocatalytic behavior with conductive nature. Furthermore, this indicates that GCC 2 have decrement of resistance and better ion transport behavior than pure Cu_2_O.

### 3.9. Photocatalytic Studies

**Figure 10:**
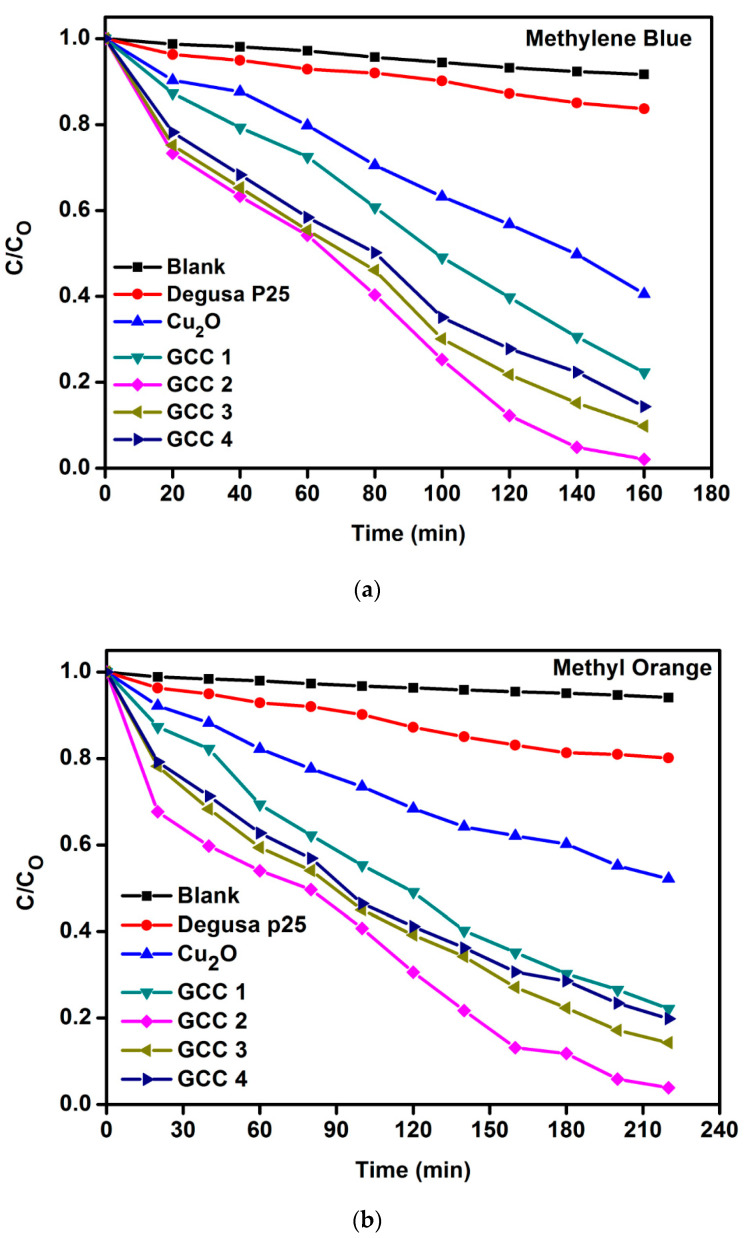
(**a**) C/C_0_ versus time for MB dye degradation. (**b**) C/C_0_ versus time for MO dye degradation.

The photocatalytic efficiencies of all GCC composites were explored against methylene blue and methyl orange dyes in aqueous conditions under visible light irradiation. Initially, the GCC is added with the dye solutions with 30 min stirring up to equilibrium attained. Here, the dye concentration should be kept as constant for all experimental conditions. The degradation of MB and MO dyes with respect to time using Cu_2_O and GCC is shown in Figure 10a,b. The absorption maxima are decreased gradually with respect to time and graphene oxide percentage. Among all composites, the GCC 2 performed highest degradation of both dyes for 160 and 220 min, respectively. The reusability of the GCC 2 nanocomposite has been checked several times, and it was observed that the degradation ability is not appreciably reduced up to three times.

**Figure 11:**
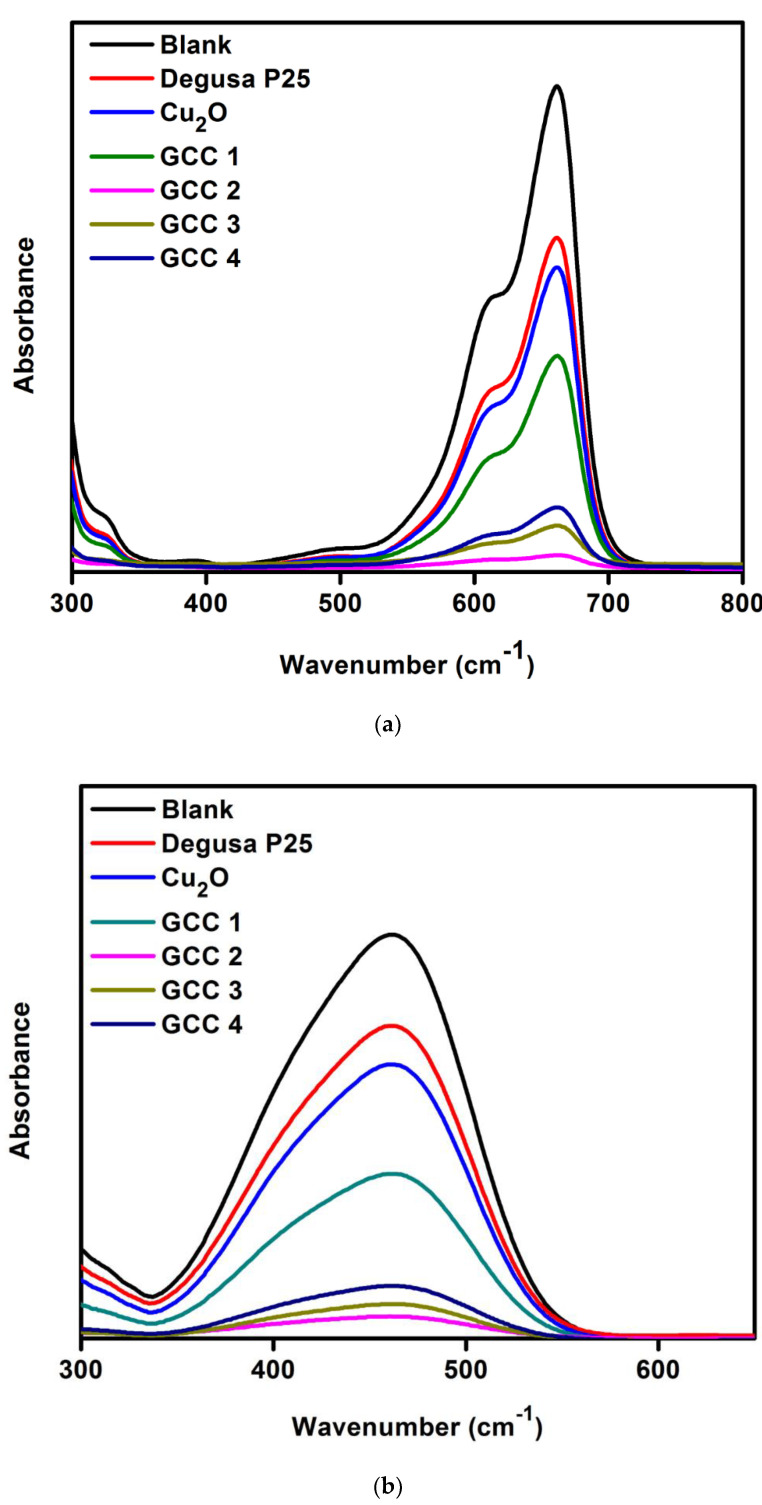
(**a**) UV spectrum of methylene blue degradation. (**b**) UV spectrum of methyl orange degradation.

Owing to the influence of visible light during catalytic process, the chromophore group’s elimination was achieved with its structural degradation. The studies of photocatalytic activities for GCC with different graphene oxide (1–4%) loadings were executed. The initial and final concentration (C/C_0_) change of MB and MO were related to maximum absorbance values. It is identified that the GCC shows highest degradation capacity than that of pure catalyst (Cu_2_O) and conventional catalyst (Degussa P25) (Figure 11a,b). MB dye degradation with GCC and Cu_2_O was found to be 97.9% and 59.44%, respectively, after 160 min of visible light irradiation. MO dye degradation with GCC and Cu_2_O was found to be 96.1% and 47.82%, respectively, after 220 min of irradiation under visible light. The percentage of degradation values are presented in Table 1.

The results of photocatalytic activities prove that amount of graphene plays a major role for the performance of GCC composites against corresponding dyes. Especially, the high surface area of graphene is able to improve the photocatalytic behavior of Cu_2_O than that of its pure form due to the reduction in electron hole pairs recombination by pi-pi* interaction involved in dye molecule. Therefore, the increasing amount of RGO has driven fast the catalytic performance of the composites. However, the degradation capacity was reduced when the GO content goes beyond the limit (i.e.,) above 2% due the shielding effect by excess GO content which confirms the influence of RGO in GCC composites.

It is observed from the degradation results that the GCC composites degrade the dyes effectively when compared to P25 catalyst and pure Cu_2_O. Among the reduced graphene oxide/Cu_2_O composites, 2% GO-loaded (GCC 2) composite reached its maximum degradation ability with 97.9% for MB and 96.1% for MO dyes under visible irradiation because of the reduction rate of recombination in Cu_2_O matrix by RGO, which facilitates the dyes degradation effectively. Furthermore, a comparative account has been made among various graphene-based nanocomposites with prepared RGO/Cu_2_O composites against MB and MO dyes as given in Table 2.

### 3.10. Kinetic Studies

**Figure 12:**
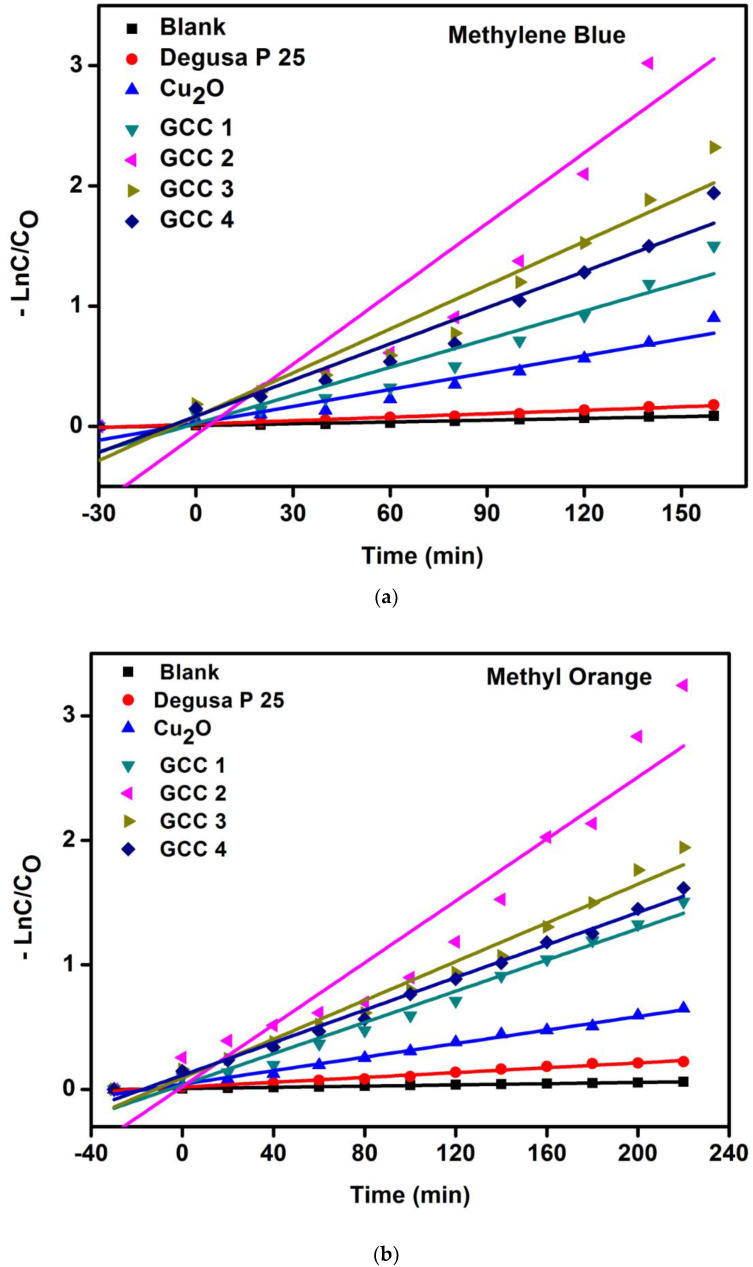
(**a**) Kinetic plot for methylene blue dye. (**b**) Kinetic plot for methyl orange dye.

The dye degradation reaction for GCC follows pseudo-first order equation by the analysis of kinetic data as shown in Figure 12a,b

−ln(C_t_/C_O_) = k_abs_ (t), (1)

where

- (i) C_O_ and C_t_—initial concentration and concentration of reactants at time ‘t’
- (ii) k_abs_ and ‘t’—apparent rate constant and time, respectively.

The various rate constant (k_abs_) values are given in Table 3, which exposes that the highest value of GCC 2 further confirms the catalytic efficiency of GCC 2 than other composites.

### 3.11. Total Organic Carbon Analysis

**Figure 13:**
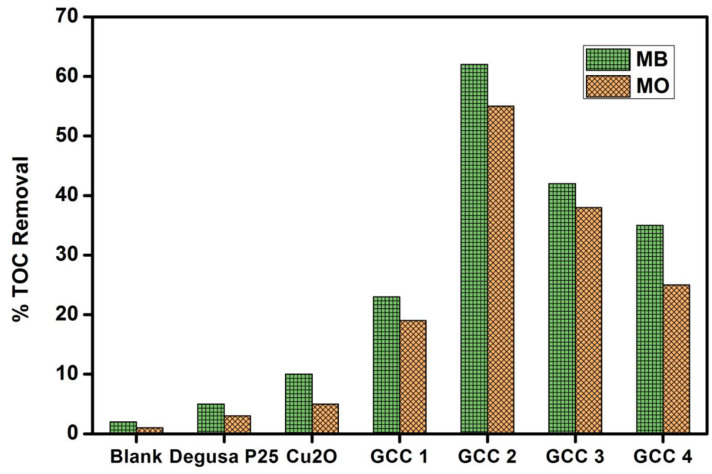
TOC removal profile for MB and MO dyes.

The degradation efficiency of the GCC composites was further confirmed by TOC analysis (Figure 13). Among the results, the higher TOC removal recorded for GCC 2 when compared with other composites, Degussa P25, and pure Cu_2_O materials. Totally, 62% of removal is attained at 160 min and 55% of removal is reached at 220 min for selected organic dyes, which further proves the degradation efficiency for GCC 2 [44,45,46].

### 3.12. Photocatalytic Mechanism

**Scheme 2:**
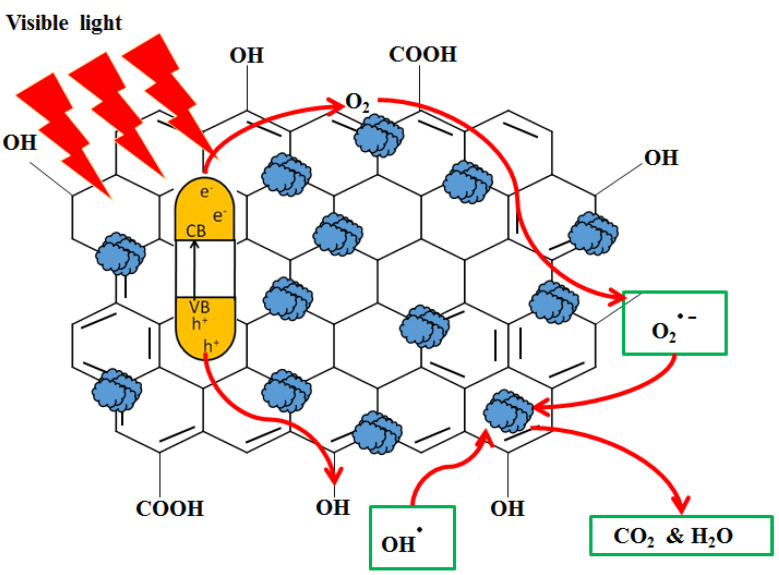
Photocatalytic mechanism of GCC.

Graphene material acts a major role in the photocatalytic mechanism of GCC catalyst, which is illustrated in Scheme 2. After the incorporation of RGO with Cu_2_O, the flow of electrons attained easily in between these two matrixes with the formation of Schottky barriers. In the meantime, the electron hole pairs are also formed for the excitement of electrons from the valence band (VB) to conduction band (CB). Then, the same electrons (e^−^) produced oxygen peroxide radicals O_2_•^−^ by reacting with the dissolved oxygen molecules. Additionally, the hydroxyl radicals OH• is obtained by treating water molecules with positively charged hole (h^+^). Finally, the organic dye molecules (MB and MO) were decomposed into CO_2_ and H_2_O molecules due the above-said free radicals.

GCC composite + Visible Light (hν) → h_VB_ ^+^ + e_CB_^−^ (2)

h_VB_ ^+^ + H_2_O → H^+^ + •OH (3)

e^−^ + O_2_ → O_2_^-^ (4)

[Dye (MB or MO)] + O_2_^−^ + •OH → H_2_O + CO_2_. (5)

From the mechanism, it is concluded that the RGO is able to reduce the recombination of electrons in between the excited to ground state, which helps to act as a better catalyst in composites for various applications especially in water treatment process.

## 4. Conclusions

The properties such as functionality, structural, and thermal and optical properties for the synthesized RGO/Cu_2_O nanocomposite were characterized by essential spectral techniques. The elemental analysis (C, O, and Cu) and morphological studies were confirmed by transmission and scanning electron microscope with EDX spectra. The RGO sheets exhibit superior interfacial contact with the Cu_2_O matrix. Further, its catalytic performance over visible region (red shift) was also enhanced to an appreciable extent, when compared with that of other samples. Hence, we can conclude that the effective GCC 2 photocatalyst will be performed as a cost-effective and ecofriendly material for the wastewater treatment in industries. The enhanced properties of the synthesized graphene-based composites will also be recommended to treat the dyes other than MB and MO in textile sector.

## Figures and Tables

**Table 1:** MB and MO degradation percentage.

S. No.	Samples	% Degradation	
		MB	MO
1	Blank	8.29	5.88
2	Degussa P25	16.41	19.87
3	Cu_2_O	59.44	47.82
4	GCC 1	77.7	77.84
5	GCC 2	97.9	96.1
6	GCC 3	90.18	85.68
7	GCC 4	85.65	80.13

**Table 2:** Comparison for degradation capacity of present work with reported photocatalyst.

Types of Graphene Metal Oxides	Methylene Blue (%)	Methyl Orange (%)	References
GO/ZnO	-	97	[30]
RGO/ZnO	90.72	89.72	[31]
GO/ZnO	100	100	[32]
GO/CdO	97	89	[33]
RGO/CuBi_2_O_4_	95	87	[34]
RGO/ZnBi_12_O_20_	96.04	94.52	[26]
GO/Ag_3_PO_4_	100	100	[35]
*NG/AgBr/CF	–	98	[36]
GO/Fe-Ti	100	–	[37]
GO/TiO_2_	–	100	[38]
GO/Pectin	98	87.5	[39]
rGO/Cu_2_O/PA	–	95	[40]
rGO/Cu_2_O	95	–	[41]
GO/MCC/Cu_2_O	83.62	–	[42]
RGO/Cu_2_O	–	98	[43]
RGO/Cu_2_O	97.9	96.1	Present work

*NG—nitrogen-doped graphene.

**Table 3:** Rate constant (k_abs_) values for the degradation process.

Samples	k_abs_ (min^−1^)						
	Blank	Degussa P25	Cu_2_O	GCC 1	GCC 2	GCC 3	GCC 4
MB	0	0	8 × 10^−3^	16 × 10^−3^	58 × 10^−3^	22 × 10^−3^	16 × 10^−3^
MO	0	1 × 10^-3^	3 × 10^−3^	5 × 10^−3^	5 × 10^−3^	6 × 10^−3^	7 × 10^−3^

## Data Availability

Not applicable.
